# The Role of the Neuromodulator Adenosine in Alcohol’s Actions

**Published:** 1997

**Authors:** Douglas P. Dohrman, Ivan Diamond, Adrienne S. Gordon

**Affiliations:** Douglas P. Dohrman, Ph.D., is a postdoctoral fellow in the Department of Neurology; Ivan Diamond, M.D., Ph.D., is professor and vice chairman in the Department of Neurology, a professor in the Department of Cellular and Molecular Pharmacology, and a member of the Neuroscience Program; and Adrienne S. Gordon, Ph.D., is a professor in the Departments of Neurology and Cellular and Molecular Pharmacology and a member of the Neuroscience Program, Ernest Gallo Clinic and Research Center, University of California, San Francisco General Hospital, San Francisco, California

**Keywords:** adenosine, neurotransmitters, receptors, neuron, cell signaling, second messenger, nucleosides, biological transport, brain, central nervous system, animal cell line, human cell line, cAMP, signal transduction, AOD intoxication, motor coordination, sleep, literature review

## Abstract

The interaction between the neuromodulator adenosine and adenosine receptors on the surface of neurons modifies the neurons’ responses to neurotransmitters. The activated adenosine receptors alter the levels of small signaling molecules (i.e., second messengers) in the cells. Depending on the receptors and cells involved, these changes can make it easier or more difficult for neurotransmitters to excite the cell. Adenosine’s activity is regulated by proteins called nucleoside transporters, which carry adenosine into and out of the cell. Alcohol interferes with the function of the adenosine system. For example, both acute and chronic alcohol exposure affect the function of the adenosine-carrying nucleoside transporters, thereby indirectly altering the second-messenger levels in the cells. Through this mechanism, adenosine may mediate some of alcohol’s effects, such as intoxication, motor incoordination, and sedation.

Alcohol abuse and dependence are among the most common and costly health problems worldwide. In the United States alone, nearly 7 percent of adults are alcohol dependent. Furthermore, the consequences of heavy drinking account for more than 20 percent of all hospitalizations (Diamond and Gordon 1997). To address the plethora of medical and social problems associated with alcohol abuse and dependence and to identify the mechanisms underlying the development of tolerance to and dependence on alcohol, it is vital to understand alcohol’s interactions with various brain systems.

One chemical that modifies (i.e., modulates) brain function and has been implicated in several of alcohol’s acute and chronic effects is adenosine. This article reviews current knowledge about alcohol’s interactions with adenosine-mediated modulation of nerve-cell activity in the central nervous system (CNS). After summarizing adenosine’s role in signal transmission in the CNS, this article discusses studies investigating alcohol’s interactions with adenosine in cell-culture (i.e., in vitro) models. Finally, the article reviews the evidence that adenosine may be an important mediator of alcohol’s effects in both animals and humans.

## Adenosine Is a Neuromodulator

Signal transmission among nerve cells, or neurons, is mediated primarily by neurotransmitter molecules that are released from the signal-emitting (i.e., presynaptic) cell and interact with specific molecules (i.e., receptors) on the surface of the signal-receiving (i.e., postsynaptic) cell.^1^ This interaction results in the excitation or inhibition of the postsynaptic cell. Adenosine also alters neuronal activity, thereby affecting behavior; however, it does not meet all the requirements for a neurotransmitter. For example, adenosine by itself does not excite or inhibit postsynaptic cells. Instead, adenosine regulates or modulates the activity of neurons in response to other neurotransmitters; it is therefore called a neuromodulator.

Adenosine is generated in all living cells during the breakdown of adenosine triphosphate (ATP), which occurs during most energy-requiring chemical reactions in the cell. Large, channel-forming proteins^2^ called nucleoside transporters then carry some of the adenosine out of the cells into the fluid surrounding the cells (i.e., the extracellular fluid). In addition, some neurons release ATP along with their neurotransmitters. This ATP also is converted into adenosine. The adenosine in the extracellular fluid can interact with receptors on the surfaces of surrounding cells (including the cell that released it) and modulate these cells’ functions. To prevent adenosine accumulation in the extra-cellular fluid and control adenosine’s effects on other cells, adenosine is taken back up into the cells by the nucleoside transporters or broken down by extracellular enzymes.

### Adenosine Receptors

**Figure 1:**
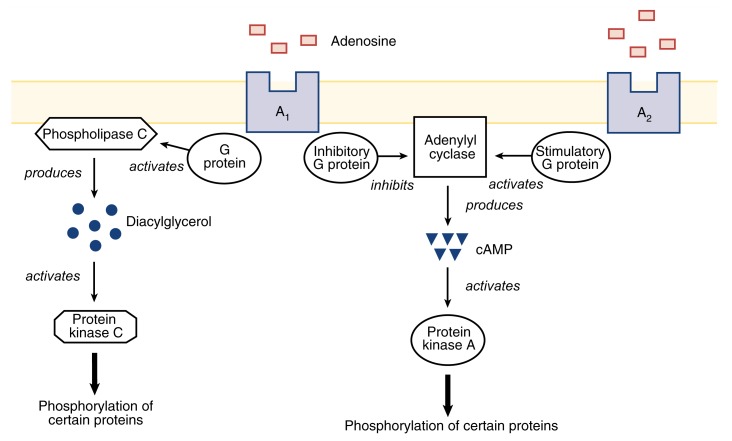
The adenosine A_1_ and A_2_ receptors affect cell function by modulating the activities of the enzymes adenylyl cyclase and phospholipase C. Through their association with inhibitory and stimulatory G proteins, A_1_ inhibits and A_2_ activates adenylyl cyclase, the enzyme that produces the second messenger cAMP. In turn, cAMP activates the enzyme protein kinase A (PKA), which adds phosphate groups to (i.e., phosphorylates) various proteins. For example, PKA phosphorylates protein channels, which allow the transport of ions across the cell membrane, and transcription factors, which alter gene activity. Phosphorylation modifies the activities of these proteins. A_1_ also activates phospholipase C, which produces the second messenger diacylglycerol. This substance activates the enzyme protein kinase C, which also phosphorylates certain proteins.

Three different subgroups of adenosine receptors exist—A_1_, A_2_, and A_3_—which differ in their structures and in the molecules with which they can interact, in addition to adenosine (for a review, see Palmer and Stiles 1995). Each cell can carry more than one type of adenosine receptor. All adenosine receptors modulate cell function primarily by altering the levels of small signaling molecules (i.e., second messengers) within the cells. Two important second messengers are cyclic adenosine monophosphate (cAMP) and diacylglycerol (DAG). These compounds activate two enzymes (i.e., protein kinase A [PKA] and protein kinase C [PKC]), which add phosphate groups to other proteins. This process is called phosphorylation. In this way, PKA and PKC activate or inactivate these proteins. Translating the activation of an adenosine receptor into changes in cell function requires multiple steps, as follows (see also figure 1):

1. The adenosine receptors are linked to regulatory molecules called G proteins. Two types of G proteins exist: stimulatory G proteins (G_s_), which enhance the activities of other enzymes, and inhibitory G proteins (G_i_), which inhibit the activities of other enzymes. The interaction of adenosine with its receptors activates G_s_ or G_i_ proteins, depending on the receptor involved.
2. Certain activated G_s_ stimulate and activated G_i_ inhibit the activity of the enzyme adenylyl cyclase, which generates cAMP.
3. cAMP activates PKA.
4. A different set of G proteins modulates the activity of the enzyme phospholipase C, which generates DAG.
5. DAG activates PKC.
6. Both PKA and PKC phosphorylate numerous proteins, including receptors for other neurotransmitters; proteins that form channels allowing charged particles (i.e., ions) to enter or leave the cell; and certain proteins called transcription factors, which regulate the activity of many genes in the cell nucleus.
7. As a result of this phosphorylation, the electrical properties of the cell change, making it easier or more difficult to excite the cell, depending on which ion channels are affected. The phosphorylation of transcription factors can cause long-term changes in gene activity that may affect cellular function.

The adenosine receptor subgroups differ in the ways in which they respond to the presence of adenosine in the extracellular fluid. Thus, A_1_ receptors can interact with adenosine even if only very low concentrations of the neuromodulator are present. Moreover, A_1_ receptors activate G_i_ (thereby inhibiting adenylyl cyclase) and modulate phospholipase C activity. As a result, protein phosphorylation in the cell is altered. In addition, activation of A_1_ receptors leads to the opening of protein channels that allow potassium ions (K^+^) to leave the cells and to the closing of calcium (Ca^2+^) channels that allow Ca^2+^ to enter the cell. As a result of these changes in ion flows, the cell becomes less excitable. (For more information on the ion flows involved in the generation of nerve signals, see the article “The Principles of Nerve Cell Communication,” pp. 107–108.) A_2_ receptors, which generally require higher adenosine concentrations to become activated, stimulate G_s_ and subsequently adenylyl cyclase, again resulting in altered protein phosphorylation. Finally, A_3_ receptors, which have been identified only recently, are coupled to G_i_ or modulate phospholipase C activity.

Researchers do not yet understand completely all second-messenger systems activated by adenosine receptors. Nevertheless, it is clear that adenosine in the extracellular fluid simultaneously activates various adenosine receptors that are associated with different second-messenger systems. These diverse interactions allow adenosine to regulate complex processes by modulating the “cross talk” between different second-messenger pathways, thereby activating or inhibiting numerous other proteins.

The adenosine receptors also differ in their regional distribution in the brain. For example, A_1_ receptors are abundant in the cerebellum, which helps control movement; the hippocampus, which is involved in memory storage; and the thalamus, which is the brain’s relay center to the cortex (Mahan et al. 1991). A_2_ receptors are most prominent in the striatum, which is involved in the programming of movements; the olfactory tubercle, which is involved in smell; the hypothalamus, which controls automatic body processes; and the nucleus accumbens, which has various functions and has been implicated in the addiction to alcohol and other drugs (Jarvis et al. 1989). However, many cells carry both A_1_ and A_2_ receptors. In these cells, adenosine’s overall effect on cell functioning likely reflects the ratio of A_1_ to A_2_ receptors. In addition, experimental evidence suggests that the same receptor type can have different effects in different areas of the same cell and that A_1_ and A_2_ receptors can be concentrated in different regions of one cell (LeVier et al. 1992). Both of these mechanisms would create additional ways for adenosine to modulate neuronal activity.

### Adenosine Modulates the Functions of Other Neurotransmitters

Adenosine affects signal transmission at many synapses that use other neurotransmitters. For example, in the peripheral nervous system, adenosine inhibits the release of the neurotransmitter acetylcholine from neurons that control the activity of muscle cells. Similarly, adenosine modulates the release of norepinephrine in the sympathetic nervous system. In the CNS, adenosine modulates the release and, in some cases, the breakdown of several neurotransmitters, including acetylcholine, serotonin, norepinephrine, dopamine, gamma-aminobutyric acid (GABA), and glutamate. For example, adenosine and adenosine agonists—compounds that mimic adenosine’s actions by binding to adenosine receptors—inhibit the release and activity of acetylcholine and the function of glutamate in the cortex (Phillis 1990; Broad and Fredholm 1996). In the striatum, adenosine agonists decrease dopamine levels (Okada et al. 1996) but increase the release of acetylcholine (Kurokawa et al. 1996). These different responses probably result from variations in the ratios and numbers of adenosine receptor subtypes on individual cells in the various brain areas.

In addition to preventing the release of neurotransmitters, adenosine can diminish the postsynaptic cell’s response to those neurotransmitters. For example, Pitchford and colleagues (1992) demonstrated that cells released adenosine when treated with a substance that activates the receptor for acetylcholine. As a result of the increase in extracellular adenosine levels, the acetylcholine receptor became less sensitive to acetylcholine binding. Thus, this receptor regulates its own function through an adenosine-mediated mechanism. Adenosine also can regulate neurotransmission by altering the strength (i.e., affinity) with which a receptor interacts with its neurotransmitter. If a receptor’s affinity decreases, higher neurotransmitter concentrations are needed to elicit the same response. In studies using membranes isolated from cells in the striatum, activation of A_2_ receptors reduced dopamine binding to a dopamine receptor called D_2_ by changing the receptor’s affinity for dopamine (Ferre et al. 1991).

## Common Characteristics of Alcohol’s and Adenosine’s Effects

Adenosine shares several functional characteristics with alcohol. Like adenosine, alcohol alters the activities of several neurotransmitter systems, inhibiting neurotransmission in some cases and enhancing it in others. Moreover, both alcohol and adenosine generally act as sedatives in the CNS. Animal studies also indicate that a correlation exists between sensitivity to adenosine and to alcohol. This observation is supported by findings that in mice, adenosine agonists exacerbate alcohol’s effects on motor incoordination. Conversely, adenosine receptor antagonists—agents that bind to adenosine receptors, thereby preventing receptor activation—ameliorate these effects. Finally, animals that are tolerant to adenosine’s effects also are tolerant to alcohol’s effects, and vice versa.

### Interactions of Adenosine and Alcohol in Cellular Models

Because of the brain’s complexity, researchers often use in vitro systems of cultured cells to examine how alcohol affects cell function. Several studies have indicated that adenosine mediates some of alcohol’s effects. One series of studies focused on two cell lines called S49 and NG108-15. These studies analyzed alcohol’s effects on the cells’ cAMP second-messenger system, on the adenosine concentrations in the extracellular fluid, and on the activity of the adenosine-carrying nucleoside transporter. (Unless otherwise indicated, the following results are reviewed in Diamond and Gordon 1997.)

#### Alcohol’s Effects on cAMP

**Figure 2:**
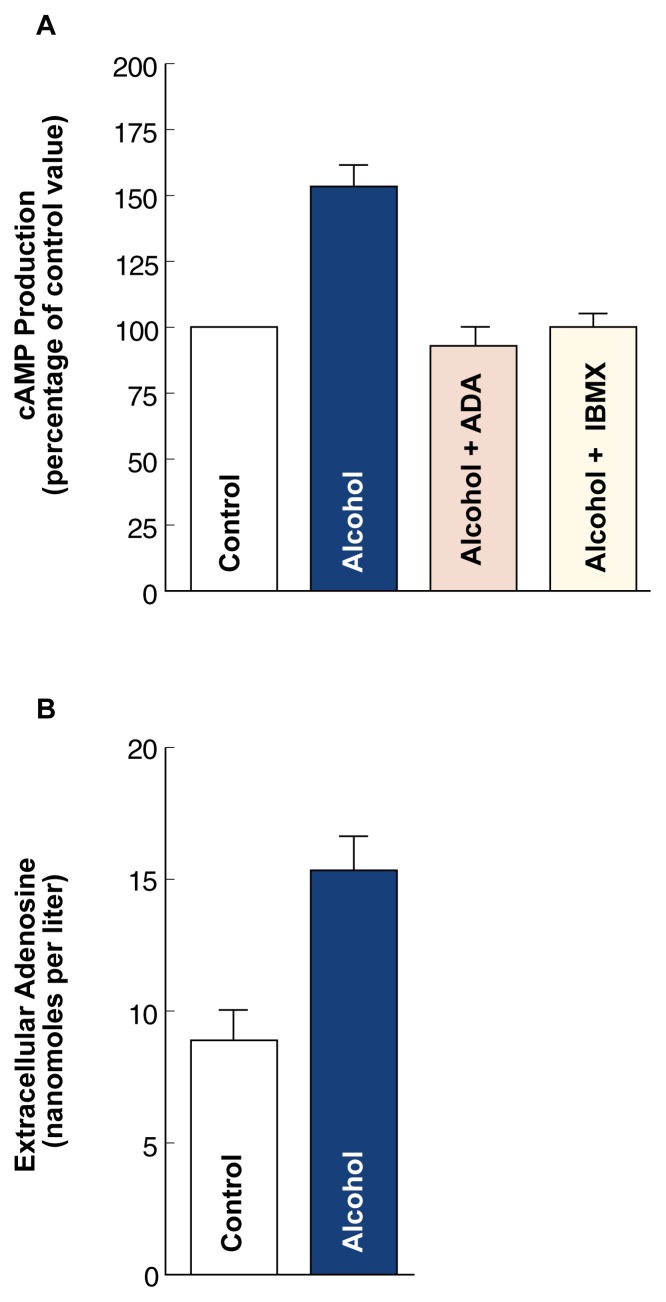
The effects of acute alcohol exposure on cAMP production and extracellular adenosine accumulation. (A) In NG108-15 cells exposed to alcohol for 10 minutes, the cAMP production increased by about 50 percent compared with untreated control cells. Addition of the enzyme adenosine deaminase (ADA), which breaks down adenosine, or of the adenosine receptor antagonist IBMX prevented the alcohol-induced increase in cAMP production. (B) When S49 cells were treated with alcohol for 10 minutes, the adenosine concentration in the growth medium almost doubled compared with untreated control cells.

When S49 or NG108-15 cells were treated for 10 minutes with alcohol at a concentration of 100 millimoles/liter (mmol/L),^3^ the cAMP levels in the cells increased by 50 percent (see figure 2). Simultaneously, the adenosine levels in the culture medium almost doubled (see figure 2). These findings suggest that short-term (i.e., acute) alcohol exposure causes an increase in extracellular adenosine. By interacting with and activating the A_2_ adenosine receptor, the excess adenosine then would lead to an increase in the intracellular cAMP levels. Adenosine’s role in the alcohol-induced increase in cAMP levels was confirmed when the cells were treated with the enzyme adenosine deaminase (ADA), which breaks down adenosine. This treatment prevented alcohol’s effects on both extracellular adenosine and intracellular cAMP. Finally, treatment of the cells with adenosine receptor antagonists also prevented the alcohol-induced increase in cAMP levels, further supporting adenosine’s role in mediating alcohol’s effects.

Whereas acute alcohol treatment stimulated cAMP production, chronic alcohol exposure caused heterologous desensitization of cAMP production in the S49 and NG108-15 cells. Desensitization means that the same stimulus (e.g., receptor activation by a certain amount of adenosine) results in a diminished response (e.g., reduced cAMP production). The term “heterologous” refers to the fact that desensitization occurs in response to all stimuli that exert their effects through a particular mechanism (e.g., through the G_s_ protein). Thus, heterologous desensitization of cAMP production in this case indicates that chronic alcohol exposure leads to reduced cAMP production not only in response to the activation of adenosine receptors but also in response to the activation of other receptors that act through the G_s_ protein. Again, the addition of ADA or adenosine receptor antagonists prevented desensitization of cAMP production. These results suggest that an increase in extra-cellular adenosine is required for alcohol-induced heterologous desensitization of receptors acting through G_s_.

In intact organisms, extracellular adenosine levels increase after alcohol consumption not only because alcohol inhibits adenosine uptake through the nucleoside transporter, but also because the breakdown (i.e., metabolism) of alcohol in the liver can result in adenosine accumulation in the blood (Carmichael et al. 1991). One product of alcohol metabolism is acetate, which can be further metabolized in a reaction that consumes ATP, thereby generating adenosine. The adenosine is released into the circulation and can thus reach the brain. Moreover, although alcohol itself cannot be metabolized in the brain, alcohol-derived acetate from the liver may reach the brain through the circulation and be metabolized there to generate adenosine. These findings suggest that the brain is directly (and indirectly through acetate) exposed to increased adenosine concentrations as a consequence of alcohol metabolism in the liver. These increased adenosine levels can exacerbate alcohol’s adverse effects on adenosine-mediated modulation of neuronal activity.

#### Alcohol’s Effect on the Adenosine-Carrying Nucleoside Transporter

The mechanism underlying the alcohol-induced increase in extracellular adenosine levels also was examined. These analyses found that acute exposure to clinically relevant alcohol concentrations (i.e., concentrations that are observed in human alcoholics) decreased adenosine uptake into the cells by 30 to 40 percent. As mentioned earlier, this transfer is performed by a specific transporter protein. Conversely, alcohol did not alter adenosine transport out of the cells. However, the reduction in adenosine uptake is sufficient to account for the extracellular adenosine accumulation observed after acute alcohol exposure. The crucial role of extracellular adenosine accumulation was underscored by observations that S49 cells altered to lack the nucleoside transporter (and which therefore do not release adenosine) showed no extra-cellular adenosine accumulation after alcohol exposure and no desensitization of cAMP production.

**Figure 3:**
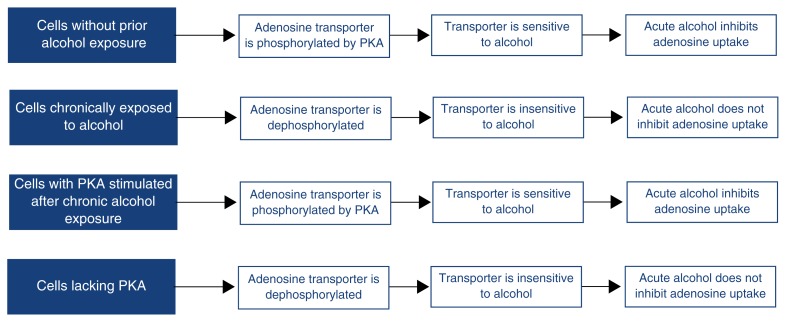
A model of the association between the phosphorylation state of the adenosine-carrying nucleoside transporter and its sensitivity to acute alcohol effects. The enzyme protein kinase A (PKA) is thought to add phosphate groups to (i.e., phosphorylate) the nucleoside transporter. Only the phosphorylated transporter is sensitive to acute alcohol exposure. If the phosphate groups are missing (i.e., the transporter is dephosphorylated), the transporter becomes insensitive to acute alcohol exposure.

When S49 and NG108-15 cells were chronically treated with alcohol, adenosine uptake was no longer inhibited (i.e., the cells became tolerant to alcohol’s inhibitory effects) (figure 3). This finding suggests that chronic alcohol exposure leads to a modification of the nucleoside transporter that renders it insensitive to inhibition by alcohol. Further analyses in NG108-15 cells suggested that this modification involved certain phosphate groups that are attached to the transporter by PKA (figure 3). Thus, Coe and colleagues (1996*a*) showed that the transporter’s sensitivity to alcohol declined when the phosphorylation by PKA declined. Furthermore, the alcohol sensitivity of cells chronically treated with alcohol was restored when the PKA activity in these cells was stimulated. Finally, a variant of the S49 cells that lacks PKA activity is insensitive to alcohol’s inhibitory effects. These data suggest that phosphorylation of the nucleoside transporter is required for its sensitivity to alcohol’s effects.

One potential mechanism underlying the decreased phosphorylation of the nucleoside transporter after chronic alcohol exposure is an alcohol-induced alteration in the location of PKA. The PKA molecules must be located near the transporter to phosphorylate it. When NG108-15 cells were chronically exposed to alcohol, however, the active part of the PKA molecule was trapped in the cell’s nucleus; consequently, PKA was unable to phosphorylate the transporter (Dohrman et al. 1996). This observation may account for the alcohol insensitivity of the nucleoside transporter in cells chronically exposed to alcohol.

**Figure 4:**
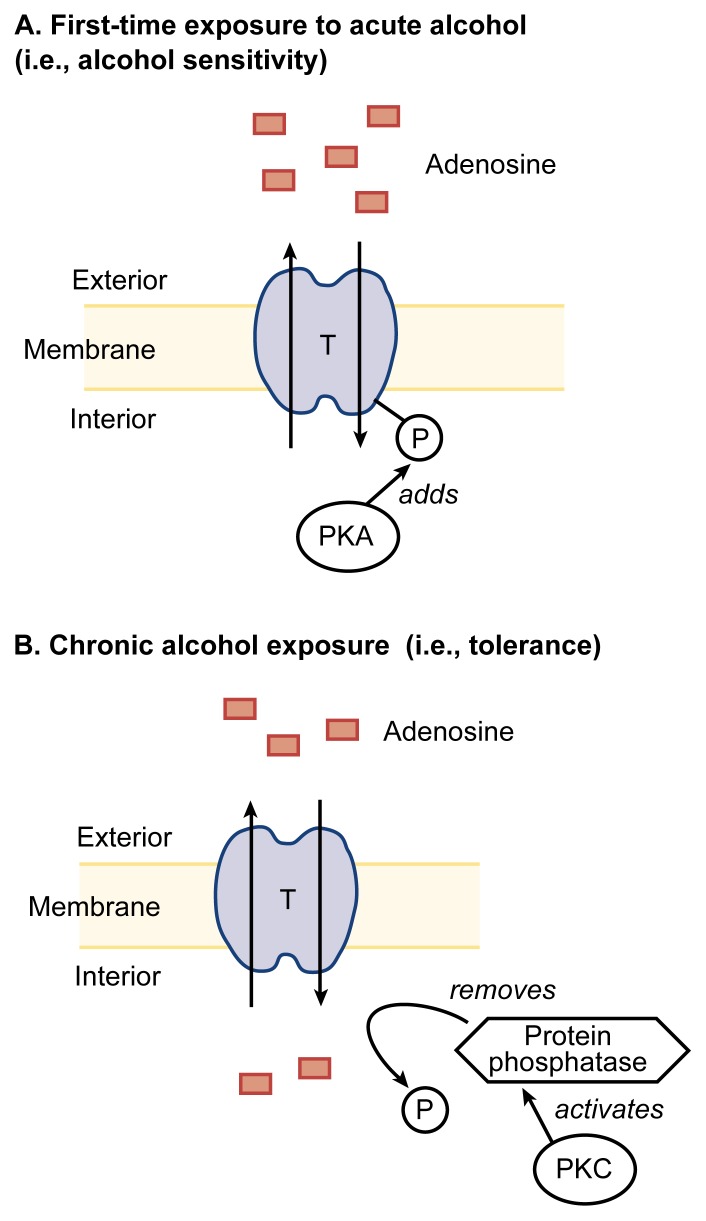
Protein kinase A (PKA) and protein kinase C (PKC) have opposing effects on the adenosine transporter’s (T’s) sensitivity to alcohol. (A) In cells that have been exposed to alcohol, PKA is thought to add phosphate groups (P) to the transporter that render it sensitive to a first-time exposure to alcohol (i.e., adenosine is not transported into the cell). (B) Conversely, after chronic alcohol exposure, PKC activates an enzyme called protein phosphatase, which removes phosphate groups from proteins, thereby rendering the transporter tolerant to alcohol’s effects (i.e., adenosine is transported into the cell even after an acute alcohol dose). This phenomenon represents an aspect of tolerance, namely, the reduced responsiveness to a previously effective drug.

The nucleoside transporter’s sensitivity to alcohol is regulated not only by PKA, but also by PKC (Coe et al. 1996*b*). However, PKA and PKC have opposite effects on the transporter’s activity. Thus, whereas PKA activity is required for the transporter’s alcohol sensitivity, activation of PKC in cells that have never been exposed to alcohol results in an alcohol-insensitive transporter (i.e., even acute alcohol exposure does not inhibit adenosine uptake). Conversely, inhibiting PKC activity during chronic alcohol exposure prevents the development of insensitivity to alcohol (i.e., tolerance). Coe and colleagues (1996*b*) have proposed a mechanism to account for PKC’s effects (figure 4). According to this hypothesis, chronic alcohol exposure increases PKC activity. PKC, in turn, activates an enzyme that removes from the nucleoside transporter the phosphate groups that have been added by PKA and which are required for alcohol sensitivity. As a result, the transporter becomes insensitive to alcohol. Together, these data suggest that PKA activity is required for alcohol sensitivity, whereas PKC activation produces tolerance. The overall activity of the adenosine transporter is determined by the balance between PKA activity and the activity of the phosphate-removing enzyme, which is regulated by PKC.

The adenosine-carrying nucleoside transporter is not the only protein related to neurotransmission whose function is regulated by phosphorylation. For example, phosphorylation by PKC may play a role in determining the alcohol sensitivity of the receptors for the neurotransmitters GABA, serotonin, and glutamate. (For more information on alcohol’s effects on these neurotransmitters, see related articles in this section.)

### Alcohol’s Effect on Adenosine Transport in Other Cell Types

Alcohol alters signal transduction not only in neurons but also in several other cell types. For example, in cultured liver cells (i.e., hepatocytes), chronic alcohol exposure decreases the amount of G_i_, thereby activating adenylyl cyclase and increasing cAMP production (Diamond and Gordon 1997). As in neurons, alcohol appears to inhibit adenosine uptake into the hepatocytes, causing an increase in extracellular adenosine. The increased extracellular adenosine levels, in turn, activate the hepatocytes’ adenosine receptors, leading to the decrease in G_i_ levels (Nagy 1994). The resulting increase in the activity of the cAMP system contrasts with the desensitization seen in NG108-15 cells. These different responses to alcohol likely can be attributed to the presence of different adenosine receptors on the cells. NG108-15 cells carry only A_2_ adenosine receptors, which act through G_s_. Hepatocytes, in contrast, carry both A_1_ receptors, which are coupled to G_i_, and A_2_ receptors. Consequently, the overall effects of alcohol-induced inhibition of adenosine uptake are determined by the type of adenosine receptors present on the cell’s surface. Because the same cell can carry both A_1_ and A_2_ adenosine receptors, the cell’s response to alcohol exposure depends on the relative amounts of both receptor types. The cell’s response also depends on the receptors’ proximity to the nucleoside transporters, which determines the adenosine concentration near the receptor. Regardless of the types of adenosine receptors present on a cell, however, the alcohol-induced inhibition of adenosine transport alters G protein-mediated signal transmission, thereby altering cell function.

### Adenosine Mediates Many Alcohol-Induced Responses in the Nervous System

Considerable evidence indicates that many of alcohol’s acute and chronic effects on the central nervous system are mediated by adenosine. For example, studies in mice have demonstrated that adenosine modulates the alcohol-induced inability to coordinate voluntary muscle movement (i.e., ataxia) (Dar 1990). This modulation may involve adenosine receptors on cells in the striatum, a brain region involved in the programming of movements (Meng and Dar 1995). Moreover, adenosine receptor agonists increase and adenosine receptor antagonists decrease alcohol-induced incoordination. Finally, a substance called dilazep, which potentiates adenosine’s effects by inhibiting adenosine uptake, exacerbates alcohol’s intoxicating effects. A role for adenosine in mediating alcohol’s effects also is supported by observations that chronic alcohol exposure induces tolerance not only to alcohol but also to adenosine antagonists, and vice versa (Dar and Clark 1992).

Other investigations of adenosine’s contribution to alcohol’s effects have used mouse strains selectively bred to differ in their sensitivity to alcohol-induced sedation. Thus, long-sleep mice experience extended sedation, whereas short-sleep mice are less sensitive to alcohol’s sedative effects. Proctor and colleagues (1985) have demonstrated that the animals’ sensitivity to alcohol correlates with their sensitivities to adenosine agonists and antagonists. These results suggest that adenosine may mediate alcohol-induced sedation in long-sleep mice.

Several other studies also have demonstrated an association between adenosine’s and alcohol’s effects on brain functioning, as follows:

- Adenosine mediates the alcohol-induced increase in the levels of β-endorphin^4^ in specific brain regions (Anwer and Soliman 1995). Accordingly, an adenosine antagonist can prevent and an adenosine agonist can enhance the alcohol-induced increase in β-endorphin.
- In tissue isolated from the hippocampus, adenosine mimics alcohol’s inhibitory effects on certain neurons (Cullen and Carlen 1992). Conversely, an adenosine receptor antagonist called 8-phenyltheophylline prevents alcohol’s inhibitory effect. Moreover, acetate has the same effects on these neurons as do alcohol and adenosine. These observations support the hypothesis that acetate mediates some of alcohol’s actions on the brain through an adenosine-dependent process (Carmichael et al. 1991).
- Alcohol concentrations of 48 mmol/L, which corresponds to blood alcohol levels of about 0.2 percent, as well as adenosine or adenosine agonists, inhibit the release of the neurotransmitter glutamate from neurons in hippocampal tissue slices (Reynolds and Brien 1995). Conversely, adenosine receptor antagonists prevent the alcohol-induced inhibition of glutamate release, suggesting that adenosine mediates alcohol’s effects on glutamate-dependent neurotransmission in the hippocampus.

## Effects of Adenosine and Alcohol on Human Cells

The effects of chronic alcohol exposure on adenosine uptake and cAMP-mediated signal transmission also have been analyzed in blood cells obtained from human alcoholics. Researchers frequently use blood cells because they are easy to obtain and may represent changes occurring elsewhere in the body (e.g., in the brain), because many signaling systems are common to different cell types. These studies found that acute alcohol exposure inhibited adenosine uptake in a type of white blood cell (i.e., lymphocytes) obtained from nonalcoholics but not in lymphocytes isolated from actively drinking alcoholics (Diamond and Gordon 1997). Similar results were obtained with preparations of membranes from red blood cells obtained from alcoholics and nonalcoholic control subjects. In addition, researchers have found that desensitization of cAMP production occurs in lymphocytes (Diamond and Gordon 1997) and platelets (Tabakoff et al. 1988) from alcoholics. Finally, chronic alcohol consumption affects the levels of G proteins in the membranes of human blood cells: The levels of G_i_ are increased in lymphocytes from abstinent alcoholics (Waltman et al. 1993), whereas the levels of G_s_ are reduced in the membranes of red blood cells isolated from actively drinking alcoholics (Nakamura et al. 1993). Although these observations do not prove that adenosine mediates these effects in alcoholics, the findings suggest that the mechanisms identified in cultured cell lines are relevant to the cellular pathophysiology of human alcoholism.

## Summary

Adenosine is a global modulator of brain activity, and substantial evidence indicates that adenosine mediates many of the acute and chronic neuronal responses to alcohol. Alcohol can increase extracellular adenosine levels both by inhibiting adenosine uptake into the cells and by increasing adenosine production throughout the body as a result of alcohol metabolism in the liver. The importance of increased adenosine concentrations is apparent at multiple levels in the nervous system. For example, altered adenosine levels affect signal transduction and gene expression in individual neurons; neurophysiological responses in the hippocampus; and behavioral responses, such as ataxia. Although adenosine clearly is not the only mediator of alcohol’s actions, it plays an important neuromodulatory role affecting the functions of several other neurotransmitters and may thus contribute to many of alcohol’s diverse effects on the brain and other organ systems in the body.

## Footnotes

1 Whereas the presynaptic cell is always a neuron, the postsynaptic cell can be either a neuron or another cell type, such as a muscle cell.

2 For a definition of this and other technical terms used in this article, see central glossary, pp. 177–179.

3 This alcohol concentration corresponds to blood alcohol levels of approximately 0.4 percent, which are sometimes seen in alcoholics after acute alcohol consumption.

4 β-endorphin is a small molecule that, like adenosine, modulates the effects of other neurotransmitters.
